# Nontoxic effects of thymol, carvacrol, cinnamaldehyde, and garlic oil on dung beetles: A potential alternative to ecotoxic anthelmintics

**DOI:** 10.1371/journal.pone.0295753

**Published:** 2023-12-20

**Authors:** José R. Verdú, Vieyle Cortez, Rocío Rosa-García, Antonio J. Ortiz, Urcesino García-Prieto, Jean-Pierre Lumaret, Carmelo García Romero, Francisco Sánchez-Piñero

**Affiliations:** 1 Research Institute CIBIO (Centro Iberoamericano de la Biodiversidad), Science Park, Universidad de Alicante, Alicante, Spain; 2 SERIDA – Servicio Regional de Investigación y Desarrollo Agroalimentario, Villaviciosa, Asturias, Spain; 3 Departamento de Química Inorgánica y Química Orgánica, Universidad de Jaén, Campus Las Lagunillas, Jaén, Spain; 4 Laboratoire Zoogéographie, Université Paul Valéry Montpellier 3, Montpellier, France; 5 Sociedad Española de Agricultura Ecológica (SEAE), Escuela Capataces Agrícolas, Catarroja, Valencia, Spain; 6 Departamento de Zoología, Universidad de Granada, Granada, Spain; Federal University of Ceara: Universidade Federal do Ceara, BRAZIL

## Abstract

The sustainability of the traditional extensive livestock sector will only be possible if healthy dung-decomposing insect communities are preserved. However, many current pharmaceutical anthelmintics are harmful to dung beetles, their presence can have a negative impact on biological systems. Phytochemical anthelmintics are an alternative to ecotoxic synthetic pharmaceutical anthelmintics, although ecotoxicological tests of their possible indirect effects on dung beetles are required to demonstrate their viability. In this study, the potential ecotoxicity of thymol, carvacrol, cinnamaldehyde and garlic oil (diallyl disulfide and diallyl trisulfide) were tested for the first time. Inhibition of antennal response was measured as a relevant parameter by obtaining relevant toxicity thresholds derived from concentration‒response curves, such as the IC_50_. All phytochemical compounds tested were demonstrated to be suitable alternative candidates to the highly ecotoxic compound ivermectin, considering their non-toxicity to nontarget organisms. Residues of the phytochemical antiparasitics found in cattle droppings were extremely low, even undetectable in the case of diallyl disulfide and diallyl trisulfide. Furthermore, our results showed that none of the phytochemical compounds have ecotoxic effects, even at extremely high concentrations, including those almost 1000 times higher than what is most likely to be found in dung susceptible to ingestion by dung beetles in the field. We can conclude that the four selected phytochemical compounds meet the requirements to be considered reliable alternatives to ecotoxic veterinary medicinal products, such as ivermectin.

## Introduction

Residues of conventional veterinary medicinal products used to treat livestock have been detected in soils, surface waters, and groundwater throughout the world [1]. Many current pharmaceutical medicinal products are harmful to invertebrate, fungal, bacterial and even vertebrate species, so their presence can have a negative impact on biological systems [2]. Studies worldwide, but especially from the Northern Hemisphere, report an alarming decline, both in abundance and diversity, of insect species, especially certain taxa such as dung beetles [3, 4]. The main factors related to this decrease in biodiversity are the chemical contamination of nontarget species by pesticides, habitat degradation (i.e., resulting from intensive practices in the livestock sector) and climate change [5].

Traditional livestock grazing practices that rely on the sustainable use of natural resources constitute key diversifying agents [6] to maintain mosaic landscapes and prevent the loss of biodiversity in agroecosystems [7]. However, traditional grazing systems have been frequently replaced in recent decades by more intensive practices, which use considerable amounts of synthetic anthelmintics as a preventive strategy to control gastrointestinal parasites in livestock.

Numerous studies have shown that the sustainability of the livestock sector will only be possible if healthy (diverse and abundant) dung decomposer insect communities are preserved because they contribute to crucial ecological activities such as dung decomposition, nutrient recycling, soil bioturbation and aeration, parasite control, plant growth, seed dispersal and changes in soil microbial communities [8–10], soil carbon sequestration in the soil [11], and a decrease in greenhouse gas emissions from dung pats [12, 13]. However, in Europe, the decline in dung beetle populations since the middle of the 20th century has been associated with the routine use of highly toxic veterinary medical products [14]. The indiscriminate use of veterinary medicinal products has been identified as the main threat to Mediterranean dung beetles, currently causing 21 endemic species from this region to be listed as threatened on the IUCN Red List [15].

Neither farmers nor related stakeholders (e.g., veterinarians) are properly informed of the adverse effects of the preventive and routine use of synthetic pharmaceutical anthelmintics on biodiversity, while the necessary alternative strategies are also limited or unknown. The decline in traditional livestock management practices concurred with the progressive loss of traditional ecological knowledge as farmers specialized and became more dependent on external inputs, including synthetic anthelmintics. Before the creation of synthetic anthelmintics in the mid-20^th^ century, farmers relied on locally available plants to control livestock intestinal parasites [16]. Thus, traditions and their associated knowledge are rapidly disappearing, with a loss of potential long-term alternatives to synthetic pharmaceutical anthelmintics [17].

Alternative plant-based products have proliferated during the past ten years as parasite resistance to traditional synthetic anthelmintics has increased [17, 18]. For example, Dr. Duke’s Phytochemical and Ethnobotanical databases from the USDA [19] compile 1,029 plants with 57 different chemical compounds with anthelmintic activity and 549 plants containing 24 chemicals with acaricide properties. Reviews also recorded more than 200 secondary metabolites (from at least ten organic chemical families) with anthelmintic activity [20, 21].

Although phytochemical anthelmintics may provide an alternative to ecotoxic synthetic pharmaceutical anthelmintics, there are no data about their possible indirect effects (once they are excreted by livestock) on nontarget organisms such as dung beetles, or even if the effects are concentration dependent. It is therefore necessary to analyze the pharmacokinetics of each phytochemical anthelmintic, measure the concentrations of the excreted residues, and assess the ecotoxicity of different concentrations of the phytochemical or its residues to coprophagous fauna.

In this study, four phytochemicals were analyzed for their suitability as true alternatives (without side effects on the dung beetle fauna) to synthetic anthelmintics: thymol (THY), carvacrol (CVR), cinnamaldehyde (CIN) and garlic oil (GAO: diallyl disulfide (DADS) and diallyl trisulfide (DATS); 1:1 ratio). THY and CVR are phenolic volatile monoterpenes present in essential oils of various Labiatae species such as *Thymus vulgaris* or *Thymus zigys*, *Origanum vulgare*, among others [22]. Previous studies verified the anthelmintic activity of THY and CVR against the nematodes *Haemonchus contortus* and *Caenorhabditis elegans* [23–27]. CIN is a phenylpropanoid compound that occurs in the bark of cinnamon trees (*Cinnamomum verum*, *Cinnamomum cassia*), showing anthelmintic activity against *Ascaris suum* and *Dactylogyrus intermedius* [28, 29]. The sulfides DADS and DATS are main components of the essential oil of *Allium sativum* [30]. Anthelmintic activity against *Schistosoma mansoni* [31], *Ascaridia galli* [32], *Haemonchus contortus*, *Moniezia expansa* [33] and Coccidia [34] has also been described for these compounds.

This study includes the first ecotoxicity tests for these alternative phytochemicals that examined both the physiological sublethal symptoms and the prelethal consequences after somatic paralysis. Following the same methodological guidelines proposed previously [35, 36], both the sensorial response of antennae (sublethal effect) and the ataxia of somatic muscles (prelethal effect) were examined by exposing a model dung beetle species (*Ateuchetus cicatricosus* (Lucas, 1846); Coleoptera, Scarabaeidae) to different concentrations of the phytochemical anthelmintics.

The objectives of the study are i) to analyze the concentrations of residues present in the feces of cattle fed a basic diet enriched with commercial alternatives according to the manufacturer’s technical recommendations; ii) to assess the toxicity of the four compounds to dung beetles through standardized ecotoxicity tests using *A*. *cicatricosus* as a model species; and iii) to evaluate the suitability of using the selected compounds as alternatives to pharmaceutical anthelmintics of proven ecotoxicity toward nontarget fauna.

## Material and methods

### Field experimental design and procedure

The experiment was conducted at Carbayal Research Station (6° 53′ W, 43° 21′ N; Sierra de San Isidro, Illano, Asturias). Nine cows of the Asturian mountain and valley breeds were used to obtain the excrement necessary to 1) analyze the excreted residues and 2) perform the ecotoxicity tests. In this experiment, we used two commercial products, one that contains a combination of THY and CVR (NEXT ENHANCE^®^ 150, Novus International Inc. USA) and another with a combination of CIN and GAO (NEXT ENHANCE^®^ 300, Novus International Inc. USA). These products are currently used as food supplements for cattle, but it is not known whether, after following the manufacturer’s technical recommendations, residues are excreted in cattle feces, their level in dung and/or their toxicity to the coprophagous fauna. The cows were weighed and stabled in different enclosures before the experiment began and assigned to the three treatments: i) three cows fed for seven days with a basal diet (5.6 kg head^–1^ day^–1^) supplemented with NEXT ENHANCE^®^ 150 (300 mg head^–1^ day^–1^); ii) three cows fed for seven days with a basal diet (5.1 kg head^–1^ day^–1^) supplemented with NEXT ENHANCE^®^ 300 (300 mg head^–1^ day^–1^); and iii) three cows fed for seven days with a basal diet only (4.5 kg head^–1^ day^–1^), as a control. NEXT ENHANCE^®^ 150 contains THY (25% wt) and CVR (25% wt) as active ingredients. NEXT ENHANCE^®^ 300 contains cinnamaldehyde (43% wt) and garlic oil (DADS + DATS; 1:1 ratio) (3.85% wt) as active ingredients. The common daily basal diet for all three treatments was herbage silage, and the ration was adjusted for each animal according to its live weight (0.5% dead matter/kg live weight). The average live weights of the cows in each treatment were 445.0 kg (control), 563.3 kg (NEXT ENHANCE^®^ 150), and 508.0 kg (NEXT ENHANCE^®^ 300). The animals had free access to water, and the total ingestion of the treatments was verified on each occasion.

Dung samples (0.5 kg) of each cow from the different treatments were collected after 1, 3 and 7 days of feeding on the experimental diets. Samples were labeled and preserved at 4 °C.

### Analysis of the chemical excreted residues

#### Fecal residue analysis: Chemicals and calibration curves

THY, CVR and CIN (purity 99%) were purchased from Acros Organics (Lancaster, UK). Standards with purity above 98% (HPLC) of major GAO components, DADS and DATS, were purchased from Sigma‒Aldrich (MO, USA). Stock standard solutions of THY and CVR were prepared in hexane at a concentration of 100 mg l^–1^. To obtain the calibration curves, solutions were prepared in the range of 20 to 0.01 ppm. To study adsorption optimization in the aqueous phase, a small amount of the stock solution was added to distilled water mixed with 10 to 30% saturated NaCl solution. Standards were prepared as follows: 3 mL of water containing 0.6 g NaCl (20%) was placed in a 10 ml glass vial closed with a magnetic and PTFE-silicone septum. Then, an accurate aliquot of stock solution was added, and the vial was heated at 25 °C in a water bath. The SPME fiber was inserted through the septum, exposing the headspace in the glass vial to collect the target hydroxylated monoterpenes at 45 °C for 60 min. The obtained samples were desorbed into a GC split/splitless injector, where desorption of the THY or CVR was carried out at 250 °C for 5 min.

#### Headspace Solid–Phase Microextraction (SPME) collection

Manure volatile collections were performed with a single field-portable SPME syringe with a 50/30 μm divinylbenzene-carboxen polydimethylsiloxane fiber (DVB/CAR/PDMS, Gray notched, Supelco, USA). The fiber was initially conditioned according to the manufacturer’s recommendations and was cleaned after each injection by keeping it in a Gas Chromatography (GC) inlet at 250 °C for 3 min.

Before volatile collection, each bottle containing a dung sample was kept for 1 h at 25 °C. Then, 60 g of manure was transferred into a clean 100 ml round glass flask (Pobel, Spain) that was immediately closed with a rubber septum cap. Each flask containing manure was heated at 45 °C in a water bath. After a 30 min solid/gas phase equilibration period, the septum of the flask was pierced using a needle, the SPME fiber was inserted, and the DVB/CAR/PDMS fiber was exposed to the headspace in the glass flask for 60 min to collect the manure volatiles. At the end of the collection period, the SPME fiber was retracted, and the needle was removed from the septum cap and introduced into the GC split/splitless injection port, where desorption of the adsorbed volatile occurred at 250 °C for 5 min.

#### Gas Chromatography-Mass Spectrometry (GC‒MS) analysis

Manure volatile analyses were carried out on a gas chromatograph (Focus GC, Thermo, USA) coupled to a single quadrupole mass spectrometer (Thermo DSQ II, USA). The MS parameters used were as follows: electron impact ionization energy 70 eV and an m/z range from 41 to 300, and the spectra were collected at 6 scans/sec. The GC was equipped with a split/splitless injection port with an SPME glass liner (1 mm ID x 120 mm straight long, ThermoFischer, USA) and a DB5 column (30 m × 0.25 mm × 0.25 μm; J&W Scientific, Folsom, CA, USA) with helium carrier gas (1.2 ml min^–1^). The injection port was maintained at 250 °C, and the SPME fiber was left extended in a splitless state for 5 min before the injector split was activated. After desorption, the fiber remained in the injector port for another 3 min. The oven temperature was programmed to start at 60 °C for 1 min, increase by 5 °C min^–1^ to 250 °C and then hold at this last temperature for 15 min. The transfer line temperature was 280 °C. Xcalibur software (Thermo Fisher) was used for data processing and reporting of GC-MS analyses. Target compounds were identified by comparing their mass spectra with those in the Wiley 275L library and matching their retention times and mass spectra to those of authentic standards.

### Ecotoxicity tests

#### Preparation of dung treatments

Cow dung without veterinary medicinal product residues was obtained from cattle used as a control in the field experiment carried out at Carbayal Research Station described above.

Two different bioassays were performed. First, an untreated control and five concentrations of THY and CVR were selected: 0.1, 1.0, 10.0, 100.0, and 1000.0 mg kg –^1^ (fresh weight), allowing us to compare a wide range of doses in a single analysis. These treatments were made by dissolving THY and CVR in absolute ethanol (Merck KGaA, Darmstadt, Germany). Then, a 2 ml aliquot of each phytochemical compound at different concentrations was added to 2 kg portions of fresh dung and subsequently mixed for 2 h with a kitchen machine mixer. Residual ethanol was removed by evaporation for 6 h before transferring the dung treatments to individual experimental units. We also added a treatment consisting of dung obtained from cows fed a basal diet with NEXT ENHANCE^®^ 150 from the field experiment. Additionally, based on previous results [35, 36], a treatment with ivermectin (IVM) at 100 μg kg –^1^ (fresh weight) was used as a positive control. This treatment was made by dissolving IVM (Merck KGaA, Darmstadt, Germany) in absolute ethanol, and a 2 ml aliquot of this treatment at 100 μg kg –^1^ was added to a 2 kg portion of fresh dung, mixing all for 2 h by means of a kitchen machine mixer. For the untreated control, absolute ethanol (2 ml) was applied to the same quantity of dung. Residual ethanol was removed by evaporation for 6 h before transferring the dung treatments to individual experimental units.

In a second bioassay, a control (noncontaminated dung), five concentrations of CIN and GAO (DADS and DATS), at the same concentrations and protocols described in the first bioassay (0.1, 1.0, 10.0, 100.0, and 1000.0 mg kg –^1^ (fresh weight)), a treatment with dung obtained from cows fed a basal diet with NEXT ENHANCE^®^ 300, and a treatment with IVM at 100 μg kg –^1^ (fresh weight) were considered. All dung treatments were placed in sealed plastic buckets to prevent desiccation during storage at 2 °C until later use.

#### Laboratory bioassay design

Each individual experimental unit consisted of a 15 × 10 × 7 cm plastic container with moist sterile paper as substrate containing one dung beetle. Experimental units were randomly assigned to the different dung treatments. Dung was supplied in 3 ml portions on a 6 cm petri dish, avoiding contact with the substrate to better quantify the amount of each phytochemical anthelmintic ingested per individual. Every three days, the unconsumed dung was removed and measured (in ml), adding a new portion of the corresponding dung treatment. In the electroantennogram (EAG) tests, beetles from each treatment were fed treated dung for an average of twenty days before conducting the bioassays. For EAG and ataxia tests, each treatment was replicated twelve times. Beetles were sexed, numbered and weighed (fresh body mass) prior to their assignment to each treatment.

#### EAG ecotoxicity test

Based on previous protocols [35, 36], electroantennogram bioassays were performed using an EAG system (Syntech, Kirchzarten, Germany) consisting of an EAG probe containing a preamplifier (Type PRG-2), a stimulus air controller (Type CS-55) and a data acquisition interface board (Type IDAC-2). The antennae of each individual *A*. *cicatricosus* were excised and mounted in an antenna holder located between two tungsten electrodes by adding small droplets of conductive gel (Spectra 360, Parker Laboratories, Fairfield, NJ, USA). The test was carried out with a constant stream of humidified air (flow of 500 ml min^−1^). A Syntech PC-based signal processing system was used to amplify and process the EAG signals. Data recorded were analyzed using EAG 2000 software (Syntech, Kirchzarten, Germany).

Stimulation tests were conducted by applying puffs of humidified air (200 ml/min) flowing for 2 s using the CS-55 stimulus controller via a Pasteur pipette containing a small strip (1 cm^2^) of filter paper (Whatman no. 1) with 1 μl of aqueous ammonia (25% NH_3_ in H_2_O, CAS Number 1336-21-6, Sigma‒Aldrich Co.) at 10%. Aqueous ammonia was used as the test compound and flow occurred through a stainless steel supply tube (1 cm diameter) with the outlet positioned approximately 1 cm from the antenna. In each experiment, the antenna was first presented with an injection of hexane (HPLC grade, Sigma‒Aldrich Co.), the standard reference compound, and then with injections of test odorants. Puffs of the tested compound were applied at 1 min intervals at least 6 times per antenna. This protocol was replicated in different individuals (n = 12, for each treatment).

#### Behavioral test

For each individual and treatment, we supplied 3 ml of dung. Based on previous protocols [35, 36], every three days, the possible negative symptoms caused by the ingestion of each phytochemical compound were evaluated by carrying out three observations: a) coordinated walking, b) reflex avoidance movements of the scape-pedicel joint of the antenna, and c) starvation. When normal behavior was observed, we concluded that the beetle was healthy. However, if partial paralysis was observed in the legs and/or antennae, we recorded the date of observation and described the symptoms. This assay was performed every three days until no symptoms were observed in the surviving individuals (final time = 20 days). Each treatment was replicated twelve times (n = 12 individuals/treatment).

### Data analyses

To determine if the concentration of the phytochemical compounds in the cow dung varied with time after administration (days 1, 3 and 7), a one-way ANOVA was performed for each treatment.

To perform the ecotoxicity test, the physiological inhibition of the antennal response was used as a standardized parameter [35, 36], interpolating the toxicity threshold (IC_50_, as the concentration of each phytochemical compound where the antennal response is inhibited by half) from concentration‒response curves. Inhibition of antennal response (% inhibition relative to EAG_control_) was calculated from EAG peak amplitude data as Inhibition (%) = 100 x ((EAG_control_ − EAG_treatment_)/EAG_control_). The concentration of each phytochemical compound that inhibited 50% of the antennal response (IC_50_) was calculated from concentration‒response curves (log (inhibitor) vs. normalized response models) fitted to the percentage of inhibition in each treatment.

In addition, since the increase in the concentration of a given chemical compound can cause a rejection of its intake, the relationship between the increase in concentration of phytochemical compounds and the amount of compound ingested by each beetle was also analyzed by means of simple linear regressions and slope tests [35, 36]. To test the potential negative effects of different concentrations of each compound (NEXT ENHANCE^®^ 150 and NEXT ENHANCE^®^ 300) on antennal response, one-way ANOVA tests were used. In each case, ivermectin treatment (IVM_100) was used as a positive control. Normality was examined using a Kolmogorov‒Smirnov test (P > 0.05 in all cases). Multiple post hoc comparisons between treatment groups against the control group were made using Dunnett’s tests (EAG test: mean of treatments < mean of control).

The software GraphPad Prism (v9, San Diego, USA) was used to perform all statistical analyses.

### Ethical approval

All experimental procedures were approved by the Ethics Committee of the University of Oviedo (Protocol number PROAE 12/2020). All methods and animal treatment used in the study were performed according to the “basic rules applicable for the protection of animals used in experimentation and other scientific purposes, including teaching” stated in RD 53/2013 of Government of Spain. The study reported in the manuscript follows the recommendations in the ARRIVE guidelines (PLoS Biol 18(7): e3000411. https://doi.org/10.1371/journal.pbio.3000411).

## Results

### Fecal residues of phytochemical compounds

Cows treated with NEXT ENHANCE^®^ 150 did not show any rejection of the food supplied every day, ingesting all of it. The average concentration of THY in the feces was 0.260 ± 0.071 mg kg –^1^, with no significant differences between times of administration (F = 0.090; d.f. = 2; P = 0.910; ANOVA). In the same dung samples, 0.038 ± 0.017 mg kg –^1^ CVR was observed, and no significant differences were found among the times of administration (F = 0.548; d.f. = 2; P = 0.604; ANOVA). In the case of cows treated with NEXT ENHANCE^®^ 300, the mean concentration of the residue was 0.129 ± 0.043 mg kg –^1^ in the case of CIN, with no significant variations among administration times (F = 0.596; d.f. = 2; P = 0.635; ANOVA). In the case of GAO, no traces of DADS or DATS were found in any of the dung samples analyzed.

### Concentration‒response curves of pure phytochemical compounds

**Fig 1:**
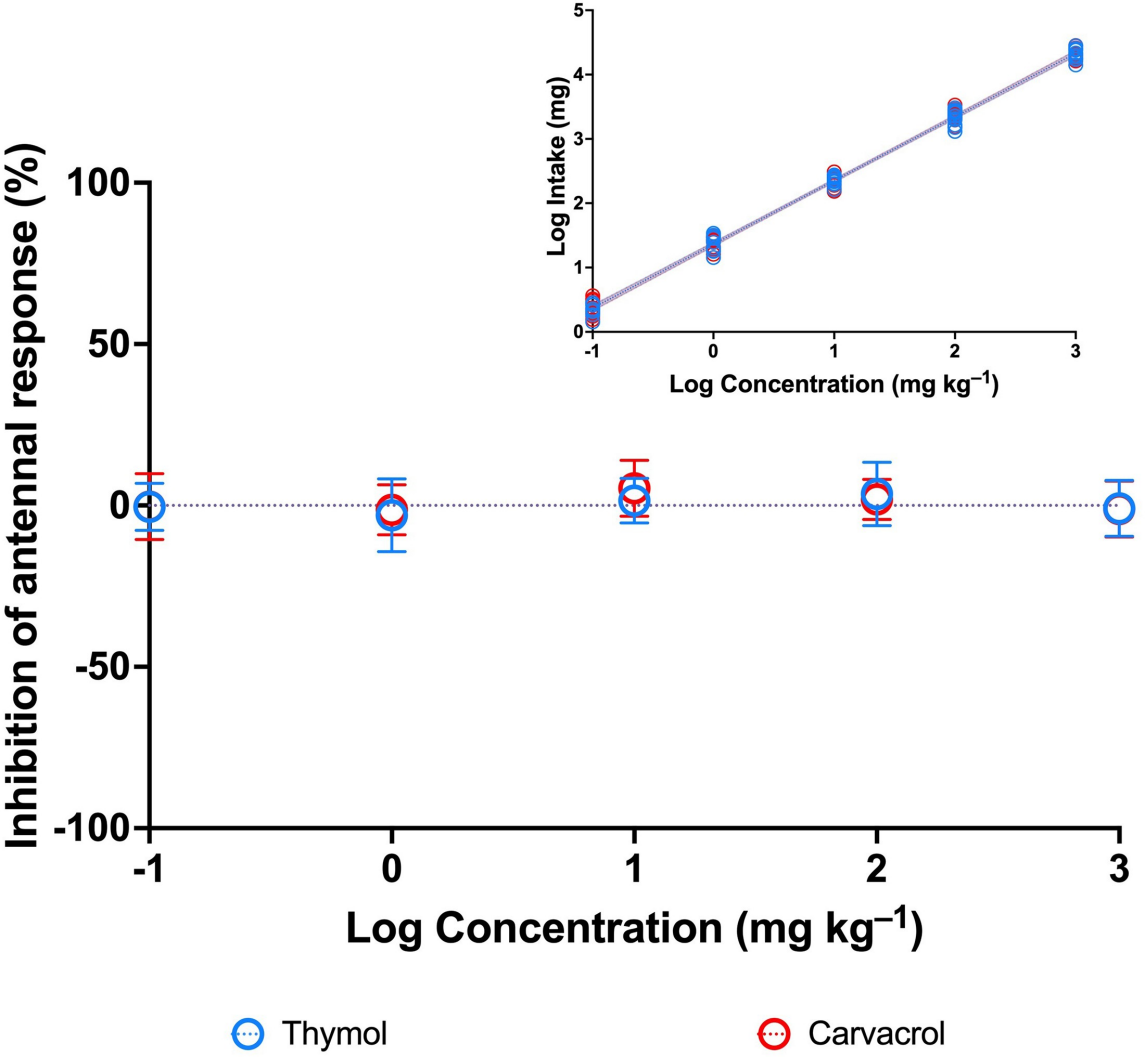
Concentration–response curves for the inhibition of antennal response by thymol and carvacrol in *Ateuchetus cicatricosus*. Bars represent ± SD (n = 12). Statistical results of the effect concentrations (IC_50_) for both phytochemical compounds are provided in Table 1. The smaller graph above shows the positive relationship between the concentrations of thymol and carvacrol and the amount of each compound ingested at the individual level.

Electroantennography recordings showed that the ingestion of THY and CVR did not affect the antennal olfactory apparatus of *A*. *cicatricosus* (Fig 1). No food rejection was observed during the bioassays, and the amount of THY and CVR ingested increased as the concentrations of each compound increased (Fig 1) (THY: F = 11903; d.f. = 1; P < 0.0001; CVR: F = 13390; d.f. = 1; P < 0.0001; simple regression). For both phytochemical compounds, the concentration‒response curves showed no relationship between the increased concentrations of THY and CVR and the antennal inhibition of *A*. *cicatricosus*. For the % inhibition of antennal response data fit with log (inhibitor) vs. normalized response models, undetermined IC50 values were observed (Table 1).

**Table 1:** Concentrations of phytochemical compounds at which the antennal response of adults of *Ateuchetus cicatricosus* was inhibited by half (IC_50_) (95% CV intervals), calculated from dose‒response curves presented in Figs 1 and 2.

Test	Thymol	Carvacrol	Cinnamaldehyde	Garlic oil
**EAG inhibition**				
IC_50_ (CI 95%) (mg kg^–1^)	n.d.	n.d.	5439 (2747–21791)	11692 (4416 –n.d.)
Goodness of Fit (d.f.; R^2^)	58; –0.00003	57; –0.001	59; –0.359	59; –0.026

n.d.: not detected.

**Fig 2:**
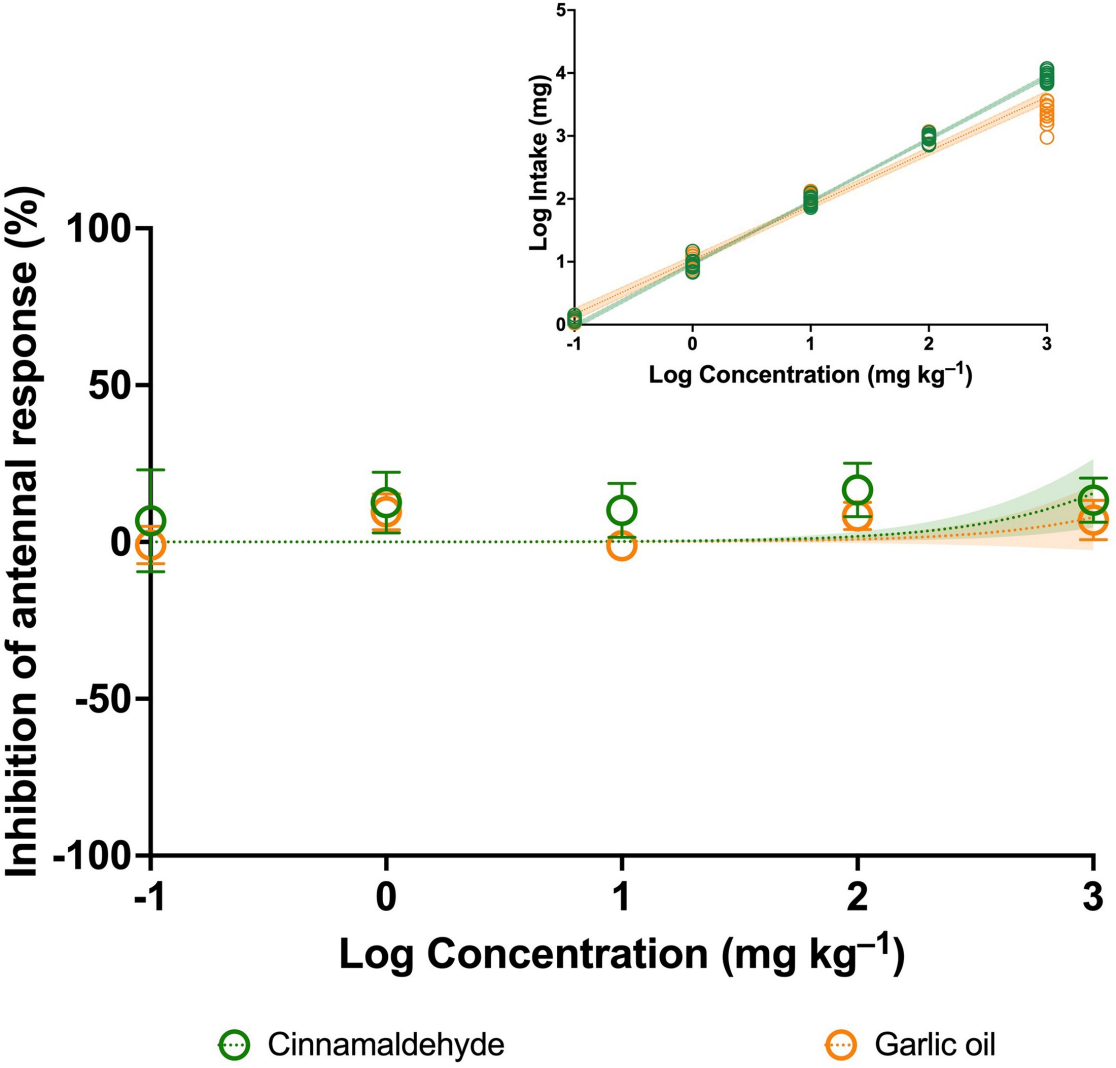
Concentration–response curves for the inhibition of antennal response by cinnamaldehyde and garlic oil in *Ateuchetus cicatricosus*. Bars represent ± SD (n = 12). Shaded areas represent the 95% confidence intervals of each model. Statistical results of the effect concentrations (IC_50_) for both phytochemical compounds are provided in Table 1. The smaller graph above shows the relationships between the concentrations of cinnamaldehyde and garlic oil and the amount of each compound ingested at the individual level.

For CIN and GAO, The EAG recordings were similar to those obtained for THY and CVR. A weak relationship between increased concentrations of CIN and GAO and antennal responses was observed, while high IC_50_ values were detected in both cases. (Fig 2 and Table 1). There was a positive relationship between the amount of compound ingested and the concentration of both CIN (F = 17518; d.f. = 1; P < 0.0001; simple regression) and GAO (F = 2078; d.f. = 1; P < 0.0001; simple regression) (Fig 2).

### Ecotoxicity of phytochemical compounds, NEXT ENHANCE^®^ 150 and NEXT ENHANCE^®^ 300 residues

**Fig 3:**
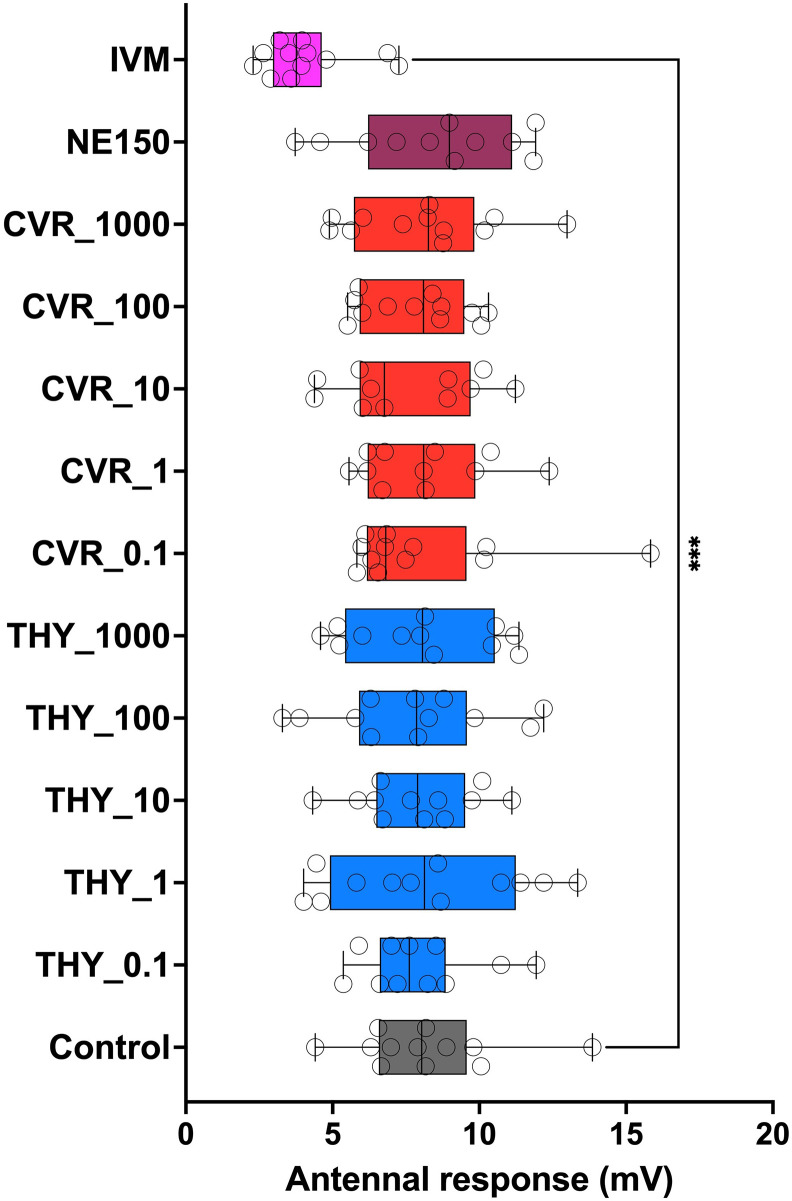
Antennal response (in mV) observed for 0.1, 1.0, 10.0, 100.0 and 1000.0 mg kg–1 thymol (THY), carvacrol (CVR) and NEXT ENHANCE 150 (NE150) compared to those observed for ivermectin at 100 μg kg^–1^ (IVM) (positive control) and for dung without any treatment (Control) (negative control). Dunn’s multiple comparisons test showed significant differences only between the control and the ivermectin treatment (*P* < 0.01).

**Fig 4:**
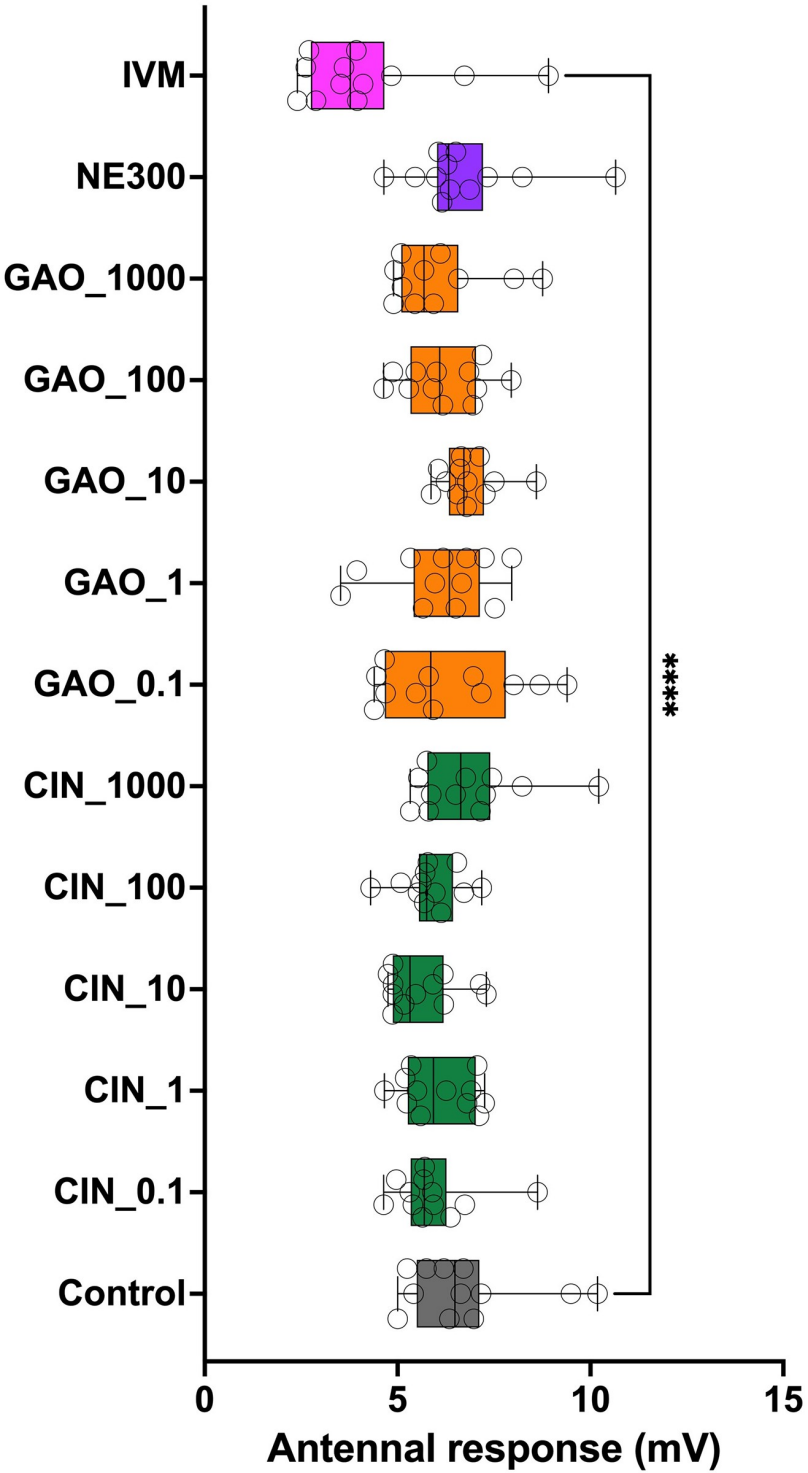
Antennal response (in mV) observed for 0.1, 1.0, 10.0, 100.0 and 1000.0 mg kg^–1^ cinnamaldehyde (CIN), garlic oil (GAO) and NEXT ENHANCE 300 (NE300) compared to those observed for ivermectin at 100 μg kg^–1^ (IVM) (positive control) and for dung without any treatment (Control) (negative control). Dunn’s multiple comparisons test showed significant differences only between the control and the ivermectin treatment (P < 0.0001).

Statistical results obtained in the first bioassay (F = 2.373, d.f. = 12; P < 0.01; ANOVA) confirmed the safety of the intake of the different concentrations of THY and CVR, as well as the NEXT ENHANCE^®^ 150 residues. Dunn’s multiple comparisons test showed significant differences only between the control and the IVM treatment (P < 0.01) (Fig 3). Similar results were obtained during the second bioassay (ANOVA: F = 3.537; d.f. = 12; P < 0.001), confirming the safety of the ingestion of CIN and GAO, even at high concentrations, as well as residues of NEXT ENHANCE^®^ 300. Dunn’s multiple comparisons test showed significant differences only between the control and the IVM treatment (P < 0.0001) (Fig 4).

During both bioassays, neither motor nor appetite symptoms indicating a negative effect of ingesting THY, CVR, CIN and GAO or either of the commercial dietary supplements NEXT ENHANCE^®^ 150 and NEXT ENHANCE^®^ 300 were observed. Negative symptoms related to the loss of motor force were observed only in 50.0% and 58.3% of the individuals fed IVM in the first and second bioassays, respectively.

## Discussion

Phytochemical compounds with anthelmintic activity, such as THY, CVR, CIN and GAO, have been demonstrated to be serious alternative candidates to highly ecotoxic veterinary medicinal products, such as IVM, toward nontarget organisms. The practical absence of chemical residues in cattle excrement after the consumption of commercial formulations as a food supplement reinforces the suitability of this type of phytochemical compounds. For THY and CVR, the extremely low concentrations of the residues in cow dung may be due to their rapid elimination through urine. For example, the half-life of THY and CVR in the digestive tract of piglets is approximately two hours due to their fast stomach and proximal intestine absorption and renal excretion [37]. In rabbits, only trace amounts of THY were detected in feces [38]. For CIN, the quantification in this matrix is complex, so the concentrations of CIN obtained in feces may be inaccurate [37]. However, it has been found that CIN is highly biodegradable [39], a result consistent with the low concentrations observed in other studies [37]. Pharmacokinetics of the major GAO components, DADS and DATS, assessing their concentrations in the stomach, plasma, liver and urine in rats showed that they are eliminated very quickly from the body [40]. This rapid elimination of the major GAO compounds could explain why no residues were found in the excrement in our experiments. The bioavailability of both major organosulfur compounds of GAO has been improved by entrapping these active compounds in microformulations, including pegylated liposomes and microemulsions [40]. Similar results were obtained with microcapsules of CIN [41] and corncob granules of THY and CVR [42]. In the case of NEXT ENHANCE^®^ 150 and NEXT ENHANCE^®^ 300, a microencapsulation system (NaturCoat^™^) is used, ensuring controlled release in the digestive tract for optimal bioavailability of active compounds.

Here, we highlight that no alternative antiparasitic should be released for organic farming unless previous standardized ecotoxicity tests confirm its safety for nontarget fauna at sublethal and lethal levels, as well as for the concentrations of residues in the excrement. For example, THY and CVR are toxic to the larval stages of the lesser mealworm beetle *Alphitobius diaperinus* (Coleoptera: Tenebrionidae) [43], the beet armyworm *Spodoptera exigua* (Lepidoptera: Noctuidae), and the planthopper *Pochazia shantungensis* (Hemiptera: Ricaniidae) [44]. CIN is toxic to the citrus flatid planthopper *Metcalfa pruinosa* (Hemiptera: Flatidae) [45], reduces food consumption and growth in larval stages of the red flour beetle *Tribolium castaneum* (Coleoptera: Tenebrionidae) [46], and has a repellent effect on the maize weevil *Sitophilus zeamais* (Coleoptera: Curculionidae) [47]. Furthermore, GAO insecticidal activity was described against the pear psyllid *Cacopsylla chinensis* (Hemiptera: Psyllidae) [48], and this compound is also toxic to the mealworm beetle *Tenebrio molitor* (Coleoptera: Tenebrionidae) [49]. Our results show that none of the phytochemical compounds have ecotoxic effects on the dung beetle *A*. *cicatricosus*, even at extremely high concentrations, including those almost 1000 times higher than what is most likely to be found in dung susceptible to being ingested by dung beetles. Thus, we can conclude that the four selected phytochemical compounds meet the requirements to be considered reliable alternatives to ecotoxic veterinary medicinal products, such as ivermectin.

These results are especially relevant given the new Regulation (EU) 2019/6 of the European Union on veterinary medicinal products. The Commission shall make a legislative proposal to introduce a simplified system for registering traditional phytochemical products used to treat animals, including the obligation to carry out pharmacological, toxicological and residue and safety tests of all chemical compounds and the obligation of taking into account scientific recommendations of the European Medicines Agency (EMA) to minimize the risk of cross-contamination and dissemination of these products in the environment as well as their unintended administration to nontarget animals [50].

## Conclusions

The findings of this study highlight the true potential of phytochemical anthelmintics to revolutionize the livestock sector. By promoting the use of these alternatives, we can significantly reduce the damage at different scales that synthetic pharmaceutical anthelmintics represent, particularly for dung beetles and the general natural balance of extensive livestock systems. Non-toxicity to non-target organisms, high safety margins and low residue levels make phytochemical compounds such as thymol, carvacrol, cinnamaldehyde and garlic oil viable options for sustainable and environmentally responsible parasite control in livestock.

The results of this study open new avenues for ecological and sustainable practices in the livestock sector, ensuring the well-being of both the industry and the environment.

Additionally, interdisciplinary studies on the effects of feed additives with new natural plant extracts with anthelmintic properties on parasite ecology and livestock health, diet selection and performance, coupled with prior standardized ecotoxicity tests, are necessary to ensure good livestock practices consistent with the Agrifood systems and the One Health vision [51].
